# Evaluation of the dietitians adherence to nutrition support guidelines or protocols in Saudi hospitals and identifications of the barriers to compliance

**DOI:** 10.3389/fnut.2025.1675530

**Published:** 2025-10-17

**Authors:** Sara Zaher, Alhanouf Sameer Alhussaini, Raneem Zuhair Abdulghani, Refal Eihab Azzouni, Seba Khalid Aalouh, Shahd Majed Alharbi, Sondos Albukhari

**Affiliations:** 1 Department of Clinical Nutrition, College of Applied Medical Sciences, Taibah University, Madinah, Saudi Arabia; 2 Department of Clinical Nutrition, Madina National Hospital, Medina, Saudi Arabia

**Keywords:** nutrition support, guidelines, adherence, enteral nutrition, parenteral nutrition

## Abstract

**Background:**

Nutrition support (NS) is essential for patients who cannot meet their nutritional needs orally. To ensure the effective implementation of nutrition care, several NS guidelines have been established to standardise practices, enhance patient safety, and improve overall clinical outcomes. This study examined adherence to NS guidelines or protocols among dietitians in Saudi hospitals and identified key barriers to compliance.

**Methodology:**

All dietitians working in hospitals across Saudi Arabia were eligible to participate in this cross-sectional study. Convenience sampling was used initially, followed by chain referral sampling, to achieve an adequate sample size. Data were collected via an online questionnaire between January and March 2025 and analysed using both univariate and multivariate analyses.

**Results:**

A total of 133 participants were included in this study. The results showed that the American Society for Parenteral and Enteral Nutrition guidelines were the most commonly used, reported by 35.6% of respondents. Participants demonstrated equally strong adherence to protocols for both enteral nutrition and parenteral nutrition, with a median adherence score of 5.00. The most frequently reported challenges to adhering to NS protocols were resistance from healthcare practitioners (60.9%), limited resources (26.2%), and poor communication with the healthcare team (23.5%). Regression analysis revealed that both hospital size (*β* = 0.732, *p* = 0.001) and the dietitians’ years of experience (*β* = −0.344, *p* = 0.007) were significant predictors of adherence level.

**Conclusion:**

This study identified several barriers and challenges to adherence to NS guidelines or protocols. To improve NS practices, strategic investments in the improvement of hospital infrastructure, the development of structured interprofessional communication frameworks, and the implementation of ongoing training programs are needed. Addressing these key areas will be essential for standardising and optimising the delivery of nutrition care in hospitals across Saudi Arabia.

## 1 Introduction

Nutrition support (NS) therapy is an alternative method to provide nutrition for patients who cannot meet their nutritional requirements orally due to medical condition (1). It plays a key role in preventing or treating malnutrition, improving clinical outcomes, reducing the length of hospital stay, and maintaining optimal nutritional status. NS is classified into two types: enteral nutrition (EN) and parenteral nutrition (PN). EN involves delivering nutrients directly into the gastrointestinal (GI) tract via tube feeding. It is indicated for patients with a functional GI tract and is commonly prescribed for individuals with dysphagia, neurological disorders, or increased metabolic demands (2, 3). Parenteral nutrition (PN), on the other hand, involves the intravenous administration of nutrients and is recommended for patients with a non-functional GI tract, such as those experiencing intestinal obstruction or severe diarrhoea (4).

Both EN and PN are complex interventions that require specialised knowledge, individualised assessment, and close monitoring to ensure safety and effectiveness (5). To ensure the effective implementation of nutrition care, several NS guidelines have been established to standardise practices, enhance patient safety, and improve overall clinical outcomes (6). These evidence-based guidelines provide structured recommendations for assessing nutritional status, determining the appropriate type of support (enteral or parenteral), selecting formulas, calculating requirements, and monitoring patient response (7, 8). In addition, standardised protocols enhance multidisciplinary collaboration and reduce variability in practice, leading to more consistent and effective nutrition care (6). Adherence to NS protocols is essential for ensuring optimal patient outcomes, particularly in hospital and critical care settings (6). Adherence to established protocols has been linked to improved clinical outcomes, reduced healthcare costs, and enhanced quality of life for patients (6). When properly followed, these protocols help maintain energy balance, preserve lean body mass, and support immune function (9). On the other hand, poor adherence to NS protocols is associated with worsened outcomes, including muscle wasting and delayed recovery from illness or surgery (10).

Despite the availability of well-established NS guidelines, significant gaps between recommended practices and their actual clinical implementation persist across hospitals and countries. These inconsistencies may be influenced by factors such as variations in institutional policies, limited access to resources, and differences in healthcare provider training. Given these challenges, this study set out to investigate dietitians’ adherence to NS guidelines or protocols in Saudi hospitals and identify the key barriers to compliance. Its findings will inform hospital administrators and policymakers about the need for improved training and resource allocation and the development of clear, evidence-based nutrition policies within Saudi healthcare institutions.

## 2 Methods

### 2.1 Study design and sampling

In this cross-sectional, survey-based study, data were collected over 2-month period from January to March 2025. All registered dietitians (RDs) employed in hospitals across various regions of Saudi Arabia and involved in the provision of NS were eligible to participate. Participants were recruited using a combination of convenience sampling and chain referral sampling to achieve an adequate sample size. The questionnaire was primarily distributed through social media platforms, including WhatsApp and X (formerly Twitter) and LinkedIn, as well as via email. The study received ethical approval from the Research Ethics Committee of the Faculty of Applied Medical Sciences at Taibah University, Madinah, Saudi Arabia [approval number 2025/207/202(CLN)]. All participants provided informed consent electronically prior to answering the questionnaire. Statements regarding confidentiality and anonymity were included on the first page of the questionnaire.

#### 2.1.1 Sample size calculation

The estimated sample size for this study was 100 participants, based on an estimated total population of 1,000 dietitians in Saudi Arabia. According to the Ministry of Health’s 2018 census, approximately 2,000 professionals are employed across various sectors within the nutrition field in Saudi Arabia. Assuming that half of them work in clinical nutrition, the number of dietitians involved in Nutrition Support (NS) is 1,000. Therefore, with a margin of error of 5% and a confidence level of 95%, the required sample size was calculated to be a minimum of 100 dietitians.

### 2.2 Questionnaire development and validation

**Figure 1:**
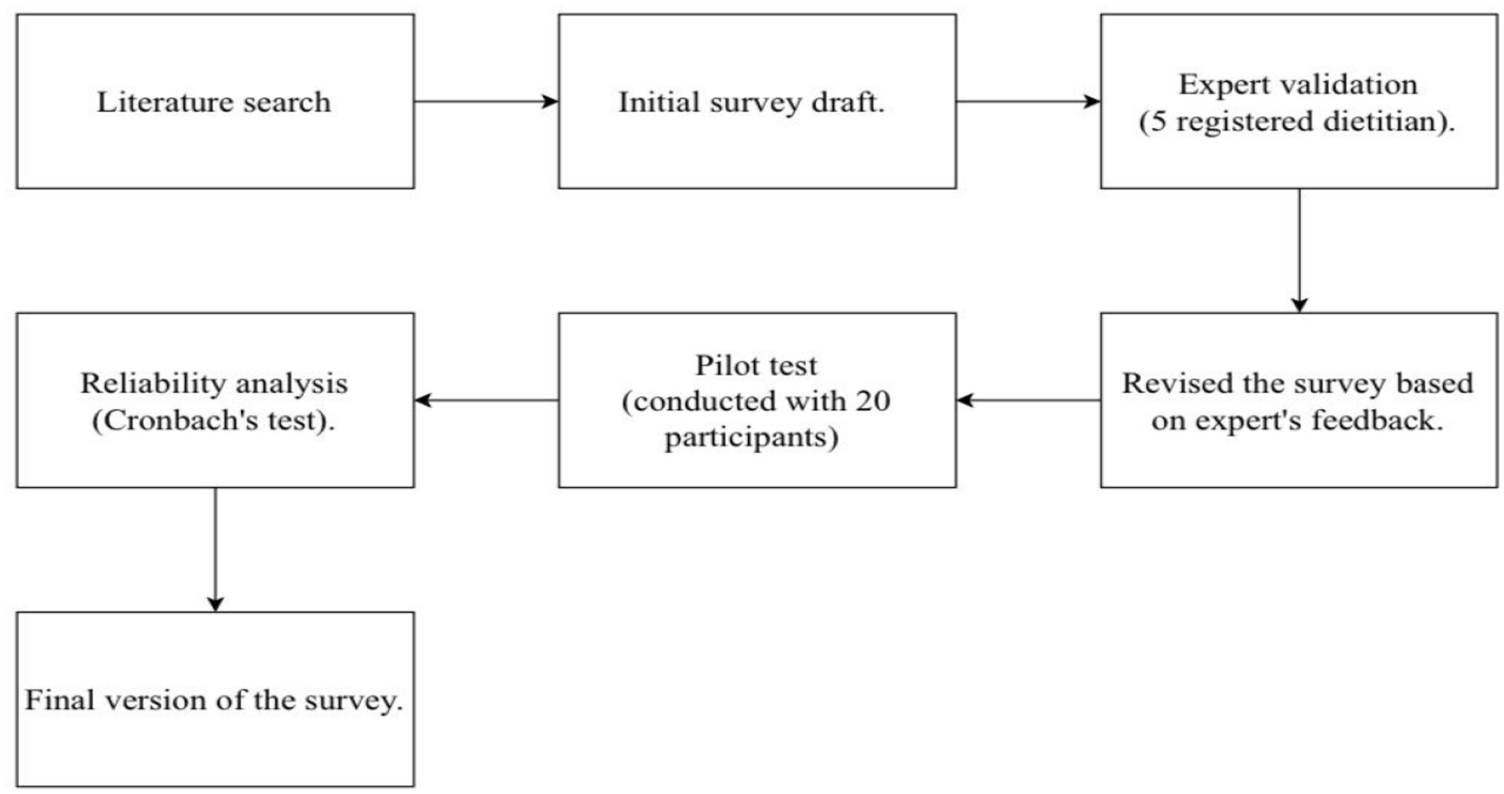
Survey development flow diagram.

The development process of the questionnaire is illustrated in Figure 1, which highlights the stepwise approach used to ensure its validity and reliability. The survey was developed by the research team following a comprehensive review of relevant literature. It underwent expert validation to ensure content accuracy and clarity, followed by pilot testing with 20 participants to assess reliability and usability. Reliability analysis was conducted using Cronbach’s alpha, leading to the final version of the survey. The revised final version of the questionnaire was then distributed electronically to dietitians working in hospitals across various regions of Saudi Arabia and directly involved in providing NS (Figure 1).

Figure 1 illustrates the step-by-step process followed in the development and validation of the survey instrument. The process began with a literature search and initial survey draft preparation, followed by expert validation by five registered dietitians. Based on expert feedback, the survey was revised and subsequently pilot-tested with 20 participants. Reliability analysis was conducted using Cronbach’s alpha, leading to the final version of the survey.

#### 2.2.1 Validation

Expert validation was carried out by five specialists in the field of clinical nutrition. The experts evaluated the survey for question clarity, simplicity, and relevance. Their feedback was thoroughly reviewed and implemented to refine the final version of the survey. Following this, the survey was pilot tested on 20 dietitians who routinely manage enteral and parenteral NS to assess its reliability and usability. The pilot test yielded a Cronbach’s alpha of 0.957, indicating excellent internal consistency.

#### 2.2.2 Description of the survey

The structured questionnaire was developed to collect data aligned with the study objectives. The first section focused on participants’ demographic characteristics and included eight questions. The second section assessed the level of adherence to NS protocols and was divided into two parts: EN, comprising nine questions, and PN, comprising seven questions. A five-point Likert scale was used to measure responses, where 1 = Never, 2 = Rarely, 3 = Sometimes, 4 = Often, and 5 = Always. The third section aimed to identify barriers to adherence to NS protocols and included four questions.

### 2.3 Statistical analysis

Following data collection, the responses underwent a thorough data cleaning process to eliminate incomplete or inconsistent entries. Subsequently, data coding was performed to prepare the dataset for statistical analysis. The cleaned and coded data were analysed using the Statistical Package for the Social Sciences (SPSS), version 23, to evaluate adherence levels and identify key barriers faced by dietitians in implementing NS protocols.

The Shapiro–Wilk test was conducted to assess the normality of the data. For normally distributed variables, the mean and standard deviation were calculated; for non-normally distributed variables, the median and interquartile range (IQR) were used. The Wilcoxon signed-rank test was used to compare related groups with non-normally distributed data.

A stepwise regression analysis was performed to identify the most significant predictors of adherence to NS guidelines or protocols. The cumulative adherence score, calculated across various components of NS practice (including screening, assessment, referral, initiation, advancement rate, formula or solution selection, and the management of NS complications), was used as the outcome variable. Independent variables included hospital size (coded as < 100 beds = 1, 100–300 beds = 2, > 300 beds = 3), primary assigned ward (medical = 1, surgical = 2, paediatric = 3, gynaecology = 4, oncology = 5, paediatric intensive care unit = 6, neonatal intensive care unit = 7, adult intensive care unit = 8, other = 9), gender (female = 1, male = 2), region (western = 1, central = 2, eastern = 3, northern = 4, southern = 5), years of experience (less than 1 year = 1, 1–5 years = 2, 6–10 years = 3, 11–15 years = 4, 16–20 years = 5), and highest academic qualification (bachelor’s = 1, residency = 2, fellowship = 3, board = 4, master’s = 5, doctorate = 6, PhD candidate = 7). All statistical tests were two-tailed, and a *p*-value of < 0.05 was considered statistically significant.

## 3 Results

A total of 209 dietitians were initially screened for eligibility. Of these, 76 were excluded as they were either not RDs (*n* = 56) or were RDs who did not provide nutrition support in their clinical practice (*n* = 20), resulting in a final sample of 133 participants included in the analysis. The majority of participants were female (74.4%), while 25.5% were male. The sample included dietitians from various regions across Saudi Arabia, with the western region representing the largest proportion (41.3%), followed by the central (33.8%), eastern (10.5%), southern (8.2%), and northern (6.0%) regions. This geographic distribution broadly reflects the demographic composition of the clinical nutrition workforce in the country. The general characteristics of the study participants are presented in Table 1.

**Table 1:** Participants’ characteristics and information.

Demographic questions	Number of dietitians (133)		
		*N*	(%)
Gender	Female	99	74.4
	Male	34	25.5
Region	Central	45	33.8
	Eastern	14	10.5
	Northern	8	6.0
	Southern	11	8.2
	Western	55	41.3
Educational level	Bachelor’s	103	77.4
	Residency	5	3.7
	Fellowship	1	0.7
	Board	1	0.7
	Masters	21	15.7
	Doctorate	1	0.7
	Other	1	0.7
Years of experience	Less than a year	41	30.8
	1–5	50	37.5
	6–10	19	14.2
	11–15	12	9.0
	16–20	11	8.2
Ward	Medical	38	28.5
	Surgical	16	12.0
	Paediatric	10	7.5
	Gynaecology	3	2.2
	Oncology	7	5.2
	Paediatric intensive care unit	5	3.7
	Neonatal intensive care unit	3	2.2
	Adults intensive care unit	43	32.3
	Other	8	6.0
Type of hospital/clinic	University teaching hospitals	4	3.0
	Specialized hospitals	18	13.5
	Private hospitals	25	18.7
	National guard hospitals	5	3.7
	Ministry Of Health (MOH) hospitals	62	46.6
	Military hospitals	9	6.7
	Medical cities	7	5.2
	Comprehensive rehabilitation center	2	1.5
	Royal Commission medical center	23	0.7
Size of hospital	Less than 100 beds	23	17.2
	100–300 beds	47	35.3
	More than 300 beds	36	47.3
Type of nutrition support	Enteral nutrition	79	59.3
	Parenteral nutrition	3	2.2
	Both	51	38.3

Data are presented as frequencies and percentages.

Percentages are based on a total of 133 participants.

Among the 284 recorded responses regarding guideline usage (as participants were allowed to select more than one guideline), the American Society for Parenteral and Enteral Nutrition (ASPEN) guidelines emerged as the most utilised, reported by 35.6% of participants, followed closely by the European Society for Clinical Nutrition and Metabolism (ESPEN) guidelines (33.5%). Internal hospital policies and protocols accounted for 28.9% of the responses. Only 2.1% of participants indicated the use of alternative guidelines (Table 2).

**Table 2:** Reported use of clinical nutrition guidelines among surveyed participants.

Guidelines	*N* = (284)*	(%)
ASPEN Guidelines	101	35.5
ESPEN Guidelines	95	33.4
Internal Hospital Policy and Guidelines	82	28.8
Other Guidelines	6	2.1

*Participants were allowed to choose more than one guideline. Percentages are based on the total number of responses (*N* = 284) from 133 participants.

ASPEN, American Society for Parenteral and Enteral Nutrition; ESPEN, European Society for Parenteral and Enteral Nutrition.

Regarding adherence to NS guidelines or protocols, participants demonstrated equally strong compliance with both EN and PN protocols, with a median adherence score of 5.00. Across all EN-related questions, the majority of the responses were “Always” ranging from 62.6% to 68.4% indicating strong adherence to policies regarding nutrient assessment, initiation and advancement, formula selection, and complication management. The median score for each EN question was 5, with a mean ranging from 4.43 to 4.49 and relatively low standard deviations, suggesting consistency across responses. Similarly, strong adherence to PN-related practices was reported, with the frequency of “Always” responses ranging from 60.8% to 66.6%. The median score for all PN-related items was also 5, while mean scores ranged from 4.25 to 4.33 (Table 3).

**Table 3:** Frequency of implementation of nutritional guidelines or protocols for enteral and parenteral nutrition in Saudi hospitals.

Questions		Never	Rarely	Sometimes	Often	Always	Mean ± SD	Median (IQR)
		(*N*) %						
EN	Q1. Does your hospital (department) implement certain policies and procedures for assessing patient’s nutrient requirements	(2) 1.6	(1) 0.8	(18) 14.6	(25) 20.3	(77) 62.6	4.44 ± 0.838	5 (1)
	Q2. Does your hospital (department) implement certain policies and procedures for enteral nutrition initiation and advancement	(4) 3.3	(3) 2.5	(12) 10.1	(21) 17.7	(78) 66.1	4.43 ± 0.965	5 (1)
	Q3. Does your hospital (department) implement certain policies and procedures for enteral formula selection	(3) 2.8	(3) 2.8	(9) 8.4	(19) 17.9	(72) 67.9	4.49 ± 0.917	5 (1)
	Q4. Does your hospital (department) implement certain policies and procedures for the management of enteral nutrition complications (e.g., aspiration, diarrhoea, etc.)	(3) 2.7	(4) 3.6	(11) 9.9	(17) 15.3	(76) 68.4	4.46 ± 0.951	5 (1)
PN	Q1. Does your hospital (department) implement certain policies and procedures for parenteral nutrition initiation and advancement	(4) 8.6	(2) 4.3	(4) 8.6	(8) 17.3	(28) 60.8	4.29 ± 1,195	5 (1)
	Q2. Does your hospital (department) implement certain policies and procedures for the selection of the type of parenteral nutrition solution (e.g., 3 in 1, 2 in 1 or ready-made bags)	(5) 12.5	(2) 5	(2) 5	(5) 12.5	(26) 65	4.25 ± 1.360	5 (1)
	Q3. Does your hospital (department) implement certain policies and procedures for the management of parenteral nutrition complications (e.g., hyperglycemia, electrolytes imbalances, etc.)	(4) 8.8	(1) 2.2	(4) 8.8	(6) 13.3	(30) 66.6	4.33 ± 1.203	5 (1)

EN, enteral nutrition; PN, parenteral nutrition.

Percentages are based on a total sample size of *N* = 284.

Participants responded using a five-point Likert scale (Never = 1, Always = 5).

The results revealed several perceived barriers and challenges affecting the dietitians’ adherence to NS guidelines or protocols. The most frequently reported barrier was limited resources, cited by 26.2% of participants. This was followed by poor communication with the healthcare team (23.5%), as a notable challenge. Specifically, when participants were asked about the frequency of resistance from other healthcare professionals, a majority (60.9%) reported encountering this issue sometimes, with a mean score of 3.02 ± 0.995, suggesting a moderate yet prevalent obstacle. Additionally, a lack of institutional support was reported by 20.2% of respondents (Table 4). The participants’ responses to queries about access to ongoing education or workshops on NS varied considerably. Only 30.8% reported that their hospitals always provide such educational opportunities, while 32.3% indicated that this occurs sometimes, and 23.3% reported that it occurs rarely. Notably, 13.5% of participants stated that they had never received any ongoing education or training related to NS.

**Table 4:** Reported challenges and barriers faced by dietitians in adhering to nutrition support guidelines or protocols.

Challenges and barriers	*N* (217)^*^	(%)
Lack of time	35	16.1
Limited resources	57	26.2
Insufficient training or education	26	11.9
Poor communication with the healthcare team	51	23.5
Lack of institutional support	44	20.2
Other	4	1.8

*Participants were allowed to select more than one challenge. Percentages are based on the total number of responses (*N* = 217) from 133 participants.

A stepwise regression analysis was then conducted to investigate the factors affecting the dietitians’ adherence to NS guidelines or protocols. The regression analysis indicated a statistically significant relationship between hospital size and adherence to the screening, assessment, and referral guideline or protocol (*β* = 0.355, *p* = 0.001). This suggests that dietitians working in larger hospitals demonstrate higher adherence levels. Other variables, including the ward assignment, gender, region, years of experience, and highest qualification, did not show statistically significant associations with adherence (*p* > 0.05). The model explained approximately 11.7% of the variance in adherence scores (*R*^2^ = 0.117; adjusted *R*^2^ = 0.109). Similarly, hospital size emerged as a significant predictor of adherence to initiation, formula selection, and complication management guidelines or protocols for EN (*β* = 0.341, *p* = 0.002), indicating better compliance among dietitians in larger hospitals. No other independent variable reached statistical significance. The model accounted for 7.9% of the variance in adherence scores (*R*^2^ = 0.079; adjusted *R*^2^ = 0.071). In the third model, we investigated the factors affecting the dietitians’ adherence to initiation, solution selection, and complication management guidelines or protocols for PN. The regression analysis showed that both hospital size (*β* = 0.732, *p* = 0.001) and years of experience (*β* = −0.344, *p* = 0.007) were significant predictors of adherence. Larger hospital size was positively associated with adherence, whereas more years of experience were associated with lower adherence. Other factors such as ward assignment, gender, region, and educational qualification did not show significant associations with adherence. This model demonstrated the highest explanatory power, accounting for 28.9% of the variance in adherence (*R*^2^ = 0.289; adjusted *R*^2^ = 0.253); see Table 5.

**Table 5:** Regression analysis to identify the factors affecting the dietitians’ adherence to nutrition support guidelines or protocols.

Adherence to screening, assessment and referral guideline or protocol of nutrition support			
Model 1 Outcome variable: cumulative score of the adhesion	*R*	*R^2^*	Adjusted *R^2^*
Dependent variable (*n* = 133)	0.341	0.117	0.109
	*β*		*p*-value
Size of the hospital^a^	0.355		0.001^*^
The ward mainly assigned to^b^	0.122		0.150
Gender^b^	−0.143		0.093
Region^b^	−0.066		0.442
Years of experience^b^	−0.003		0.975
Highest certificate earned^b^	−0.110		0.210

Adherence to guidelines or protocols of EN initiation, formula selection, and complication management			
Model 2 Outcome variable: cumulative score of the adhesion	*R*	*R^2^*	Adjusted *R^2^*
Dependent variable (*n* = 133)	0.281	0.79	0.071
	*β*		*p*-value
Size of the hospital^a^	0.341		0.002*
The ward mainly assigned to^b^	0.145		0.096
Gender^b^	−0.137		0.114
Region^b^	0.021		0.814
Years of experience^b^	−0.111		0.208
Highest certificate earned^b^	0.105		0.245

Adherence to guidelines or protocols of PN initiation, solution selection, and complication management			
Model 3 Outcome variable: cumulative score of the adhesion	*R*	*R^2^*	Adjusted *R^2^*
Dependent variable (*n* = 44)	0.537	0.289	0.253
	*β*		*p*-value
Size of the hospital^a^	0.732		0.001*
Years of experience^a^	−0.344		0.007*
The ward mainly assigned to^b^	0.190		0.167
Gender^b^	−0.195		0.177
Region^b^	0.130		0.339
Highest certificate earned^b^	0.198		0.164

a Predictors (constant).

b Excluded variables.

*R^2^*: Coefficient of determination.

*Significant at *p* < 0.05.

## 4 Discussion

The current study provides an overview of dietitians’ adherence to NS protocols or guidelines in Saudi hospitals and the factors influencing their compliance. The majority of the participants were female. The findings revealed that ASPEN and ESPEN guidelines were the most frequently utilised, with a significant proportion of participants also relying on internal hospital protocols. The dietitians demonstrated high and consistent adherence to both EN and PN protocols. However, several challenges and barriers to consistent adherence were identified, including poor communication with healthcare teams and resistance from other healthcare professionals, limited resources, and inadequate institutional support. The current study also showed that participants working in bigger hospitals had higher compliance scores. Finally, more years of experience were associated with lower adherence scores, particularly in the management of PN nutrition.

This study indicated that the ASPEN and ESPEN guidelines were the most frequently utilised in Saudi Hospitals. This reflects the global influence of these organisations, which provide evidence-based, comprehensive guidelines that address a wide range of clinical conditions, in the field of clinical nutrition (11). The variability observed in the use of these two guidelines among participants may be largely attributed to differences in their educational and training backgrounds. Dietitians who received their professional education in institutions or regions where a particular guideline is emphasized are more likely to adopt and consistently apply that guideline in their clinical practice. Previous studies have similarly reported variability in the use of growth charts among dietitians, which was partly linked to differences in their academic training. This is particularly relevant in the Saudi context, where curricula across health disciplines and educational institutions can vary considerably, leading to differences in exposure to specific tools and guidelines. Moreover, beyond formal education, healthcare professionals refine their skills and preferences through clinical experience, which can further shape their choice and consistent use of specific guidelines in practice (12). A significant proportion of our sample also indicated reliance on internal hospital protocols. This aligned with the findings from another study indicating that several hospitals have developed and implemented their own NS guidelines (13). Overall, our findings highlight the predominance of the ASPEN and ESPEN guidelines, while the noteworthy reliance on internal hospital protocols suggests variability in guideline adoption across institutions. Moreover, the absence of nationally enforced unified nutrition support protocols has been highlighted as a critical gap in the healthcare system, as it may hinder the consistency of clinical practice and limit opportunities for quality improvement initiatives (14). Further research is needed to explore how healthcare institutions in Saudi Arabia and similar settings adapt these guidelines into locally relevant protocols. Understanding the extent and nature of this institutional adaptation is critical, as it may directly influence the consistency, feasibility, and quality of NS practices across different hospital environments. Such investigations could provide valuable insights into potential gaps between guideline recommendations and their real-world application.

The current study identified several barriers and challenges to the consistent adherence to nutrition support (NS) guidelines and protocols. Among the most frequently reported obstacles was resistance from other healthcare professionals, which participants reported as a key factor limiting protocol implementation. This finding aligns with previous national and international research documenting similar patterns of resistance. For example, studies have reported reluctance among clinicians to initiate early EN in intensive care settings and a lack of acceptance of dietitian-led recommendations in clinical practice (15, 16). Likewise, a scoping review on multidisciplinary nutritional care for hospitalized adults highlighted that poor collaboration and opposition from certain healthcare team members can significantly impede the implementation of nutrition protocol (15). Further evidence suggests that communication gaps, unclear role boundaries, and bureaucratic barriers also contribute to these challenges (16). In the Saudi Arabian healthcare context, several studies have documented similar challenges. For instance, Alsoqeah et al. (17) reported that RDs in Riyadh encountered barriers such as limited resources and insufficient institutional support including the absence of standardized protocols and Lack of opportunities for continuing professional education. While our study quantified adherence levels and linked them to factors such as hospital size and years of experience, the qualitative insights provided by Alsoqeah et al. (17) offer valuable depth by illustrating how these barriers are experienced in day-to-day practice. Likewise, Aldubayan et al. (18) found that physicians in Riyadh demonstrated low to moderate knowledge of clinical nutrition particularly regarding nutrition support therapy and the nutrition care process further supporting our finding that knowledge gaps among physicians may contribute to interprofessional resistance and limit effective collaboration with dietitians. These findings indicate that interprofessional collaboration challenges, insufficient institutional support, and limited resources remain significant barriers in some clinical settings, hindering the consistent implementation of nutrition support (NS) protocols. However, it is important to emphasize that these barriers should not be interpreted as reflections of the overall quality or international standing of Saudi hospitals. Rather, they represent operational and organizational obstacles that may vary across institutions due to differences in internal policies, departmental coordination, and the availability of continuing education opportunities. Previous research has likewise shown that even in well-resourced healthcare systems, variability in institutional policies and shortcomings in interprofessional collaboration can hinder the consistent application of evidence-based nutritional practices—underscoring the critical role that internal institutional policies play in facilitating or impeding adherence to NS protocols (19) Taken together, these findings highlight the need for unified national standards and system-level strategies, integrated within the medical accreditation framework, to further support consistent adherence to nutrition support protocols.

Our findings revealed a statistically significant association between hospital size and adherence scores to NS guidelines or protocols, indicating that dietitians working in larger hospitals had higher levels of compliance. This may be attributed to the greater resource availability, more efficient infrastructure, and better organisational support typically found in larger healthcare institutions. Moreover, such hospitals often have the financial capacity to invest in ongoing staff training and specialised nutrition education, which can further promote adherence to evidence-based protocols. These results align with previous research showing that larger hospitals are more likely to implement formalised NS programs, which facilitate protocol-driven care and contribute to improved patient outcomes. The structured systems and multidisciplinary collaboration commonly present in larger facilities likely play a key role in enabling more consistent and effective NS practices (20–22). Thus, in the present context, the level of adherence to NS guidelines among dietitians is likely shaped by structural and policy-related factors specific to the Saudi healthcare system. The healthcare sector in Saudi Arabia is largely centralised and governed by the Ministry of Health alongside other major providers such as military hospitals, medical cities, and private institutions. While international guidelines such as those from ASPEN and ESPEN are widely referenced and utilised, there is currently no nationally unified protocol for NS mandated across all healthcare facilities. This absence of standardisation contributes to variability in clinical practice and may hinder the consistent implementation of evidence-based nutrition care. It is also worth noting that the vast majority of participants in this study were employed in public hospitals, with only a small proportion working in private hospitals or centers. This distribution likely limited our ability to capture potential differences in nutrition support practices across healthcare settings. To address these gaps, further research is needed to explore the systemic factors influencing guideline adherence, including institutional policies, professional roles, and organisational culture. In addition, studies with more balanced representation of public, private, and specialized institutions are warranted to further investigate how institutional context may influence adherence to nutrition support protocols. Such research can inform national policymaking and support the development and enforcement of standardised NS protocols across healthcare institutions. As part of a national strategy to improve consistency and adherence, it is recommended that NS standards be incorporated into hospital accreditation processes, such as those governed by the Saudi Central Board for Accreditation of Healthcare Institutions. This would ensure that compliance with evidence-based nutrition practices becomes a measurable and enforceable criterion within healthcare quality frameworks.

The majority of participants in this study were early-career dietitians with 5 years or less of professional experience (68%), which may explain the overall high adherence rates observed across most nutrition support protocols. Notably, the association between greater years of experience and lower adherence was detected only in the regression analysis for PN management, rather than across all protocol domains. However, the relationship between a dietitian’s experience level and protocol adherence is complex and influenced by various factors. For instance, while more experience can foster confidence in clinical abilities, this heightened confidence does not necessarily correlate with better adherence to protocols. Our findings on experienced dietitians having lower adherence rates align with a prior study by Vo et al., which found that experienced dietitians with substantial clinical exposure often develop robust clinical judgment that they prioritise over protocol adherence (23) Another perspective is that dietitians who entered the field earlier may have less exposure to the latest evidence-based guidelines compared to more recently trained peers. In addition, long-practising dietitians may not update their practices as thoroughly, resulting in lower protocol adherence (11). This may be further complicated by the fact that real-world applications, especially in complex settings, often require personalised adjustments that experienced dietitians feel more capable of making based on their expertise (24). In contrast, less experienced dietitians may rely more heavily on structured protocols to guide their clinical decision-making. This reliance on established protocols is particularly evident among practitioners who have not yet developed the extensive clinical intuition that sometimes leads experienced dietitians to deviate from protocol-based treatments. Moreover, adherence to protocols by less experienced dietitians can be seen as part of their ongoing professional development. They are often more engaged with continuing education and quality improvement initiatives that emphasise evidence-based practice, as discussed by Ajabnoor et al. (11). This observation is further supported by an Australian cross-sectional survey in which 28 dietitians managing adult patients with head and neck cancer were assessed for their awareness, agreement, adoption, and adherence regarding national evidence-based nutritional guidelines. The study reported that dietitians with fewer years of clinical experience (less than 10 years) demonstrated higher adherence scores to these guidelines compared with more experienced colleagues, particularly regarding the use of structured nutrition protocols (25) Experienced dietitians, on the other hand, may be more influenced by their clinical experience, leading to modified practices or protocol drift, where adaptations to individual patient contexts occur at the potential expense of strict adherence. This interpretation remains speculative, and future research, particularly qualitative studies, is recommended to explore the reasons underlying this finding.

The current study has several notable strengths. The use of a mixed-methods approach incorporating both quantitative data and qualitative insights through the exploration of the dietitians’ practices regarding the utilisation of NS guidelines enabled a nuanced understanding of not only the extent of protocol adherence but also the contextual factors influencing clinical nutrition practices. To enhance the generalisability of the findings, the study included participants from a variety of hospital types across multiple geographical regions in Saudi Arabia. While several studies in the region have investigated NS practices (11, 26–31), to our knowledge, this is the first study to specifically assess the level of dietitians’ adherence to NS protocols or guidelines. This research serves as an important initial step toward understanding current practices and promoting the standardisation of evidence-based nutritional care within the healthcare system. However, the study was limited by the self-reported nature of the data collected, which may introduce response bias, although it is important to note that self-reporting is a common approach in studies assessing dietetic practices in clinical settings. To enhance data credibility and minimise bias, participants were asked to provide their institutional email addresses to verify their professional identities. Additionally, the study sample was predominantly female; however, this trend is consistent with global and regional patterns in the field of dietetics. This gender distribution can be attributed to the feminised nature of the profession, which is often associated with caregiving and nutrition, fields that traditionally attract more women. The United Nations Development Programme has described the role of the nutritionist as a feminised profession. In Colombia, 92.7% of dietitians are female; in Chile, the proportion is 90.6%; and in Canada, over 95% of RDs are women (11). Similar patterns are observed in the Arab region. A regional study conducted during the COVID-19 pandemic across five countries (Lebanon, Saudi Arabia, Kuwait, Oman, and Tunisia) found that female dietitians made up 83.1% of the sample (30). In this study, participants were recruited using a combination of convenience and chain referral sampling to achieve an adequate sample size. While this approach may introduce bias and limit external generalisability, it was considered appropriate for the study context as it enabled the recruitment of participants who shared the key characteristics of interest, thus improving representativeness within the defined target community.

## 5 Conclusion

This study evaluated current dietetic practices regarding adherence to clinical guidelines or protocols in Saudi hospitals and identified key barriers to compliance. Adherence to NS protocols is essential for ensuring timely and adequate nutritional care, which plays a vital role in promoting patient recovery, improving disease outcomes, and reducing hospital length of stay. However, several challenges, such as limited resources, competing clinical priorities, resistance from other healthcare professionals, poor interprofessional communication, and a lack of institutional support, often compromise the effective implementation of these protocols. This study also demonstrated that adherence to NS protocols was influenced by factors such as hospital size and dietitians’ years of experience. These findings highlight the importance of promoting consistent, evidence-based nutrition care across diverse hospital settings and practitioners with varying levels of clinical experience. Furthermore, this study offers valuable insights for policymakers and healthcare administrators to develop targeted strategies that strengthen dietetic services and improve interdisciplinary collaboration. Given the strong link between protocol adherence and improved patient outcomes, addressing these issues is essential to enhancing NS quality in hospitals.

## Data Availability

The datasets presented in this article are not readily available because of confidentiality reasons to protect the participants. Requests to access the datasets should be directed to the corresponding author, Sara Zaher.
