# Correction: Evaluation of the dietitians adherence to nutrition support guidelines or protocols in Saudi hospitals and identifications of the barriers to compliance

**DOI:** 10.3389/fnut.2025.1730501

**Published:** 2025-12-23

**Authors:** Sara Zaher, Alhanouf Sameer Alhussaini, Raneem Zuhair Abdulghani, Refal Eihab Azzouni, Seba Khalid Aalouh, Shahd Majed Alharbi, Sondos Albukhari

**Affiliations:** 1 Department of Clinical Nutrition, College of Applied Medical Sciences, Taibah University, Madinah, Saudi Arabia; 2 Department of Clinical Nutrition, Madina National Hospital, Medina, Saudi Arabia

**Keywords:** nutrition support, guidelines, adherence, enteral nutrition, parenteral nutrition

In the published article, reference 11 was erroneously inserted in both the **Results** and **Discussion** sections during production, which led to changes in the citation order within the **Discussion** section. The correct sentence reads as follows, “This reflects the global influence of these organisations, which provide evidence-based, comprehensive guidelines that address a wide range of clinical conditions, in the field of clinical nutrition (11).”

In the published article, reference 12 was erroneously inserted at the beginning of the **Discussion**, paragraph 2 in the following sentence, “This reflects the global influence of these organisations, which provide evidence-based, comprehensive guidelines that address a wide range of clinical conditions, in the field of clinical nutrition (12).” It should have been inserted at the end of the following sentence, “Moreover, beyond formal education, healthcare professionals refine their skills and preferences through clinical experience, which can further shape their choice and consistent use of specific guidelines in practice (12).”

In the published article, reference 12 was erroneously inserted in the Discussion, paragraph 4 in the following sentences, “In addition, long-practising dietitians may not update their practices as thoroughly, resulting in lower protocol adherence (12)” and “They are often more engaged with continuing education and quality improvement initiatives that emphasise evidence-based practice, as discussed by Ajabnoor et al. (12).” The correct reference number should be (11).

In the published article, reference 12 was erroneously inserted in the Discussion, paragraph 5 in the following sentence, “The United Nations Development Programme has described the role of the nutritionist as a feminised profession. In Colombia, 92.7% of dietitians are female; in Chile, the proportion is 90.6%; and in Canada, over 95% of RDs are women (12).” The correct reference number should be (11).

In the published article, reference 13 was erroneously inserted in the Discussion in the following sentence, “Moreover, beyond formal education, healthcare professionals refine their skills and preferences through clinical experience, which can further shape their choice and consistent use of specific guidelines in practice (13).” It should have been inserted at the end of the following sentence, “This aligned with the findings from another study indicating that several hospitals have developed and implemented their own NS guidelines (13).”

In the published article, reference 14 was erroneously inserted in the **Discussion** in the following sentence, “This aligned with the findings from another study indicating that several hospitals have developed and implemented their own NS guidelines (14). It should have been inserted at the end of the following sentence, “Moreover, the absence of nationally enforced unified nutrition support protocols has been highlighted as a critical gap in the healthcare system, as it may hinder the consistency of clinical practice and limit opportunities for quality improvement initiatives (14).

In the published article, reference 15 was erroneously inserted in the **Discussion**, paragraph 2 in the following sentence, “Moreover, the absence of nationally enforced unified nutrition support protocols has been highlighted as a critical gap in the healthcare system, as it may hinder the consistency of clinical practice and limit opportunities for quality improvement initiatives (15).” It should have been inserted in the **Discussion**, paragraph 3 at the end of the following sentence,” For example, studies have reported reluctance among clinicians to initiate early EN in intensive care settings and a lack of acceptance of dietitian-led recommendations in clinical practice (15, 16).”

In the published article, reference 16 was erroneously inserted in the **Discussion** in the following sentence, “Likewise, a scoping review on multidisciplinary nutritional care for hospitalized adults highlighted that poor collaboration and opposition from certain healthcare team members can significantly impede the implementation of nutrition protocol (16).” The correct reference at the end of this sentence should be (15) and it should have been inserted in the following sentence, “Further evidence suggests that communication gaps, unclear role boundaries, and bureaucratic barriers also contribute to these challenges (16).”

In the published article, reference 17 was erroneously inserted in the **Discussion** in the following sentence, “Further evidence suggests that communication gaps, unclear role boundaries, and bureaucratic barriers also contribute to these challenges (17).” It should have been inserted in the following two sentences, “For instance, Alsoqeah et al. (17) reported that RDs in Riyadh encountered barriers such as limited resources and insufficient institutional support including the absence of standardized protocols and Lack of opportunities for continuing professional education. While our study quantified adherence levels and linked them to factors such as hospital size and years of experience, the qualitative insights provided by Alsoqeah et al. (17) offer valuable depth by illustrating how these barriers are experienced in day-to-day practice.”

In the published article, reference 18 was erroneously inserted in the **Discussion** in the following sentences, “For instance, Alsoqeah et al. (18) reported that RDs in Riyadh encountered barriers such as limited resources and insufficient institutional support including the absence of standardized protocols and Lack of opportunities for continuing professional education. While our study quantified adherence levels and linked them to factors such as hospital size and years of experience, the qualitative insights provided by Alsoqeah et al. (18) offer valuable depth by illustrating how these barriers are experienced in day-to-day practice.” It should have been inserted in the following sentence, “Likewise, Aldubayan et al. (18) found that physicians in Riyadh demonstrated low to moderate knowledge of clinical nutrition particularly regarding nutrition support therapy and the nutrition care process further supporting our finding that knowledge gaps among physicians may contribute to interprofessional resistance and limit effective collaboration with dietitians.”

In the published article, reference 19 was erroneously inserted in the **Discussion** in the following sentence, “Likewise, Aldubayan et al. (19) found that physicians in Riyadh demonstrated low to moderate knowledge of clinical nutrition particularly regarding nutrition support therapy and the nutrition care process further supporting our finding that knowledge gaps among physicians may contribute to interprofessional resistance and limit effective collaboration with dietitians.” It should have been inserted in the following sentence, “Previous research has likewise shown that even in well-resourced healthcare systems, variability in institutional policies and shortcomings in interprofessional collaboration can hinder the consistent application of evidence-based nutritional practices—underscoring the critical role that internal institutional policies play in facilitating or impeding adherence to NS protocols (19).”

In the published article, reference 20 was erroneously inserted in the **Discussion**, paragraph 3 in the following sentence, “Previous research has likewise shown that even in well-resourced healthcare systems, variability in institutional policies and shortcomings in interprofessional collaboration can hinder the consistent application of evidence-based nutritional practices—underscoring the critical role that internal institutional policies play in facilitating or impeding adherence to NS protocols (20).” It should have been inserted in the **Discussion**, paragraph 4 in the following sentence, “The structured systems and multidisciplinary collaboration commonly present in larger facilities likely play a key role in enabling more consistent and effective NS practices (20–22).”

In the published article, reference 11 was erroneously referenced as “Hill, LT. Nutrition support practices in south African ICUs: results from a nationwide pilot survey. South Afr J Crit Care. (2015) 31:42–50. doi: 10.7196/SAJCC.2015.v31i2.252”. The correct reference reads as follows, “Ajabnoor SM, Zaher S, Malatani R, Jawa H. Exploring the practice of nutritional support during hospitalization across physicians, dietitians, and pharmacists based in Saudi Arabia. *Front Nutr*. (2023) 10:1149727.”

The corrected reference list is below:

The original article has been updated.
