# Supplementary material for: Evaluation of the dietitians adherence to nutrition support guidelines or protocols in Saudi hospitals and identifications of the barriers to compliance
Source: Front Nutr. 2025 Oct 17;12:1675530. doi: 10.3389/fnut.2025.1675530 (PMC12587766; doi:10.3389/fnut.2025.1675530)
Supplement: Supplementary file 1 [file Data_Sheet_1.PDF]

Evaluation Of Dietitians Adherence to Nutrition Support Protocols in Saudi Hospitals and identifications of The Barriers to Compliance: A Survey-Based Study

Consent form

Thank you for your interest in participating in this survey. Please review the consent form below and decide if you want to participate in the study.

Dear prospective participant,

Researchers from Taibah University, Clinical Nutrition Department invite you to participate in this survey about enteral nutrition complications in the intensive care settings.

You are invited to participate in this study if you are a **registered dietitian working in Saudi Hospital**

1. What is the study about?

This study aims to evaluate the adherence of dietitians in Saudi hospitals to established nutrition support protocols and identify the key barriers that hinder their compliance. The findings of the study are intended to improve the quality of nutrition support services in Saudi hospitals by addressing a critical gap in understanding dietitian adherence to established protocols.

2. Who can participate?

All dietitians working in public and/or private hospitals across Saudi Arabia.

3. What is expected from you?

After you give your consent, you will complete the survey to answer questions about the demographic information and the level of adherence to nutrition support protocols as well as the barriers to compliance with these protocols or guidelines. The survey is expected to take about 2 minutes to complete.

4. What are your rights and responsibilities?

Your participation in this survey is completely voluntary. You have the right to refuse to participate or stop the survey at any time after given your consent. Your refusal or stopping the survey after you started will not have any consequences. If you stop the survey before completing it, you agree to let us use the answers you provided up to that point. Your responses will be anonymous, and your identity will not be identified during any part of this research. This study was approved by the ethics committee of the University of Taibah.

5. What are the reasons you might choose to volunteer to participate in this study?

Although you might not get personal benefits from taking part in this research, your responses will help us to learn about the level of adherence to nutrition support protocols as well as the barriers to compliance. This will provide feedback and direction toward optimizing the nutrition care in Saudi Hospitals.

Contact details

If you have any questions or comments regarding this survey, you can contact information is given below:

Dr. SaraA. Zaher B.Sc. (Clin.Nut) M.Sc,PhD (paediatrics & critical care nutrition)
Associate professor
Clinical Nutrition Department, Faculty of Applied Medical Sciences, Taibah University
E.mail: Szaher@taibahu.edu.sa

6. Giving consent

- I have read the consent form, and I agree to participate in this survey
- I do not agree to participate in this survey

Section 1: Demographic Questions:

1. **Are you a registered dietitian working in Saudi Hospital?**
    - Yes
    - No (If the answer is no, the survey will end)
    -

2. **Please specify the setting**
    - Medical ward
    - Surgical ward
    - Paediatric ward
    - Gynaecology ward
    - Oncology ward
    - Paediatric intensive care unit
    - Neonatal intensive care unit
    - Adult intensive care unit
    - Other (Please specify……………………..)

3. **What type of nutrition support do you handle in your ward?**
    - Enteral Nutrition
    - Parenteral Nutrition
    - Both
    - None (If the answer is none, the survey will end)

4. **Please select your gender**
    - Male
    - Female

5. **How long have you worked in your current role?**

- Less than a year
- 1-5 years
- 6-10 years
- 11-15 years
- 16-20 years

6. **What is the highest degree that you have earned?**
    - Bachelor's
    - Residency
    - Fellowship
    - Board
    - Master's
    - Doctorate
    - Other (Please specify……………………..)

7. **Do you have additional Certification?**

   - Yes
   - No

8. Please list your certification

   ----

9. **What is the region you are based in?**
   - Western.
   - Central.
   - Eastern.
   - Northern.
   - Southern.

10. **What type of hospital/clinic do you work in?**
    - University teaching hospitals
    - Specialized hospitals
    - Private hospitals
    - National guard hospitals
    - Ministry Of Health (MOH) hospitals
    - Military hospitals
    - Medical cities
    - Other

11. **What is the size of your hospital?**
    - Less than 100 beds
    - 100–300 beds
    - More than 300 beds

<u>Section 2: Adherence to Nutrition Support Protocols Questions:</u>

<u>Part I: Screening and assessment</u>

12. **Which of the following guidelines for nutrition support are you using as a reference in your institution? (Choose all that apply)**
    - ASPEN guidelines
    - ESPEN guidelines
    - Internal hospital policy and guidelines
    - None
    - Other (Please specify ………………………………….……………)

13. **Does your hospital (department) implement certain policies and procedures for screening ?**

    - Yes
    - No **<u>(If the answer is No, will skip question 15)</u>**

14. **On a scale from 1 (Never) to 5 (Always), How often do you follow the Screening protocols in your daily practice?**
    - Always
    - Often
    - Sometimes
    - Rarely
    - Does your hospital (department)
    - implement certain policies and
    - procedures for screening ?

15. **Does your hospital (department) implement certain policies and procedures for assessing patient's nutrient requirements?**
    - Yes
    - No **(If the answer is No, will skip question 17)**

16. **On a scale from 1 (Never) to 5 (Always), How often do you follow the assessment of patient's requirements protocols in your daily practice?**
    - Always
    - Often
    - Sometimes
    - Rarely
    - Does your hospital (department)
    - implement certain policies and
    - procedures for screening ?

Part II: Enteral Nutrition

17. **Does your hospital (department) implement certain policies and procedures for enteral nutrition initiation and advancement?**
    - Yes
    - No **(If the answer is No, will skip question 19)**

18. **On a scale from 1 (Never) to 5 (Always), How often do you follow the enteral nutrition initiation and advancement protocols in your daily practice?**
    - Always
    - Often
    - Sometimes
    - Rarely
    - Never

19. **Does your hospital (department) implement certain policies and procedures for enteral formula selection?**
    - Yes
    - No **(If the answer is No will skip question 21)**

20. **On a scale from 1 (Never) to 5 (Always), How often do you follow the enteral formula selection protocols in your daily practice?**
    - Always
    - Often
    - Sometimes

- Rarely
- Never

21. Does your hospital (department) implement certain policies and procedures for the management of enteral nutrition complications (e.g. aspiration, diarrhoea, ..etc.) ?

- Yes
- No (**<u>If the answer is No will skip question 23)</u>**

22. On a scale from 1 (Never) to 5 (Always), How often do you follow the enteral nutrition management protocol in your daily practice?

- Always
- Often
- Sometimes
- Rarely
- Never

<u>Part III: Parenteral Nutrition</u>

23. Do you handle Parenteral Nutrition in your ward?

- Yes
- No **<u>(If the answer is No, will go to section 3)</u>**

24. Does your hospital (department) implement certain policies and procedures for parenteral nutrition initiation and advancement?

- Yes
- No **<u>(If the answer is No, will skip question 26)</u>**

25. On a scale from 1 (Never) to 5 (Always), How often do you follow the parenteral nutrition initiation and advancement protocols in your daily practice?

- Always
- Often
- Sometimes
- Rarely
- Never

26. Does your hospital (department) implement certain policies and procedures for the selection of the type of parenteral nutrition solution (e.g. 3 in 1, 2 in 1 or ready-made bags)?

- Yes
- No (**<u>If the answer is No will skip question 28)</u>**

27. On a scale from 1 (Never) to 5 (Always), How often do you follow the parenteral nutrition solution selection protocol?

- Always
- Often
- Sometimes
- Rarely

- Never

28. **Does your hospital (department) implement certain policies and procedures for the management of parenteral nutrition complications (e.g. hyperglycaemia, electrolytes imbalances, ..etc.) ?**
    - Yes
    - No (**If the answer is No will skip question 30)**

29. **On a scale from 1 (Never) to 5 (Always), How often do you follow the parenteral nutrition management protocol in your daily practice?**
    - Always
    - Often
    - Sometimes
    - Rarely
    - Never

Section 3: Barriers to Compliance with Nutrition Support Protocols Questions:

30. **What challenges do you face when adhering to nutrition support protocols? (Select all that apply)**
    - Lack of time
    - Limited resources
    - Insufficient training or knowledge
    - Poor communication with the healthcare team
    - Lack of institutional support
    - Other (Please specify……………………..)

31. **How often do you encounter resistance from other healthcare professionals when implementing protocols?**
    - Always
    - Often
    - Sometimes
    - Rarely
    - Never

32. **Do you feel that the current protocols are practical and applicable to your patients?**
    - Yes, always
    - Sometimes
    - Rarely
    - No

33. **Does your hospital provide ongoing education or workshops on nutrition support?**
    - Yes, always
    - Sometimes
    - Rarely
    - Never

34. **Do you have any additional comments or suggestions to improve adherence to nutrition support protocols?**
………………………………………………………………………………………………………
………………………………………………………………………………………………………

35. **Please add your institutional e-mail for future communication**

Submit the survey
